# Engineering 3D Scaffold‐Free Nanoparticle‐Laden Stem Cell Constructs for Piezoelectric Enhancement of Human Neural Tissue Formation and Function

**DOI:** 10.1002/advs.202310010

**Published:** 2024-07-25

**Authors:** Emma Claire James, Eva Tomaskovic‐Crook, Jeremy Micah Crook

**Affiliations:** ^1^ ARC Centre of Excellence for Electromaterials Science Intelligent Polymer Research Institute AIIM Facility University of Wollongong Fairy Meadow NSW 2519 Australia; ^2^ Arto Hardy Family Biomedical Innovation Hub Chris O'Brien Lifehouse Camperdown NSW 2050 Australia; ^3^ School of Medical Sciences Faculty of Medicine and Health The University of Sydney Camperdown NSW 2006 Australia; ^4^ Institute of Innovative Materials AIIM Facility Innovation Campus Faculty of Engineering and Information Systems University of Wollongong Fairy Meadow NSW 2519 Australia

**Keywords:** 3D, barium titanate nanoparticles, electrical stimulation, electroceuticals, human neural stem cells, human neural tissues, piezoelectric, ultrasound

## Abstract

Electrical stimulation (ES) of cellular systems can be utilized for biotechnological applications and electroceuticals (bioelectric medicine). Neural cell stimulation especially has a long history in neuroscience research and is increasingly applied for clinical therapies. Application of ES via conventional electrodes requires external connectors and power sources, hindering scientific and therapeutic applications. Here engineering novel 3D scaffold‐free human neural stem cell constructs with integrated piezoelectric nanoparticles for enhanced neural tissue induction and function is described. Tetragonal barium titanate (BaTi03) nanoparticles are employed as piezoelectric stimulators prepared as cytocompatible dispersions, incorporated into 3D self‐organizing neural spheroids, and activated wirelessly by ultrasound. Ultrasound delivery (low frequency; 40 kHz) is optimized for cell survival, and nanoparticle activation enabled ES throughout the spheroids during differentiation, tissue formation, and maturation. The resultant human neural tissues represent the first example of direct tissue loading with piezoelectric particles for ensuing 3D ultrasound‐mediated piezoelectric enhancement of human neuronal induction from stem cells, including augmented neuritogenesis and synaptogenesis. It is anticipated that the platform described will facilitate advanced tissue engineering and in vitro modeling of human neural (and potentially non‐neural) tissues, with modeling including tissue development and pathology, and applicable to preclinical testing and prototyping of both electroceuticals and pharmaceuticals.

## Graphical abstract

Human neural tissues are engineered from 3D scaffold‐free stem cell constructs with integrated piezoelectric (BaTiO3) nanoparticles for ultrasound‐mediated electrical stimulation during development. Stimulation throughout the constructs increases neural tissue function and maturation. The platform is scaleable and potentially amenable to engineering human neural tissues more representative of in vivo mature tissues, for advanced tissue modeling, therapy, and electroceuticals.

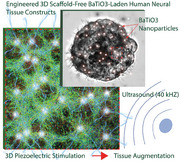

## 1 Introduction

Human neural stem cells (hNSCs) have extended capacity for self‐renewal and multi‐lineage potential, including the ability to differentiate into neurons, oligodendrites, and astrocytes in response to specific stimuli.^[^ ^1^ ^]^ Subsequently, they hold great potential for regenerative medicine strategies to treat traumatic brain and spinal cord injury and prominent neurological conditions such as Parkinson's disease, amyotrophic lateral sclerosis, stroke, and epilepsy.^[^ ^2^ ^]^ Additionally, hNSCs are amenable to in vitro modeling of neural development, cell physiology, disease phenotypes, and optimization of in vivo neural interface devices and pharmaceutical development.^[^ ^3^ ^]^

Recently, exogenous electrical stimulation has received considerable attention for artificially influencing cellular or tissue behaviors in vitro.^[^ ^4^ ^]^ All living cells and tissues within the human body produce naturally occurring bioelectric potentials arising from the asymmetrical segregation of charged ions and biomolecules across the plasma membrane.^[^ ^5^ ^]^ Intra‐ and extracellular electric fields initiate intrinsic signaling pathways and influence the intercellular microenvironment, thus driving cellular activities such as migration, proliferation, differentiation, regeneration, and cellular communication networking.^[^ ^6^ ^]^ Recent studies have shown that exogenous electrical stimulation can emulate endogenous electric fields for augmented maturation and fate determination during cell differentiation.^[^ ^4^, ^7^ ^]^ More specifically, electrical stimulation of cultured hNSCs favors neuronal over neuroglial induction with concomitant enhanced neurite outgrowth and neurogenesis.^[^ ^8^ ^]^

The application of exogenous electrical stimulation via conventional electrodes requires external connectors and power sources, significantly hindering clinical translation. For example, invasive techniques, such as deep brain stimulation, require penetrating electrodes to particular brain regions. These approaches employ wire‐based electrodes that perforate the skull, thus creating an inherent risk for infection, inflammation, and chronic gliosis.^[^ ^9^ ^]^ Other neural stimulation techniques such as transcranial direct current stimulation, transcranial magnetic stimulation, and vagus nerve stimulation are less invasive but typically have a relatively lower spatial resolution, leading to nonspecific stimulation of brain regions adjacent to the target area.^[^ ^10^ ^]^

**Figure 1:**
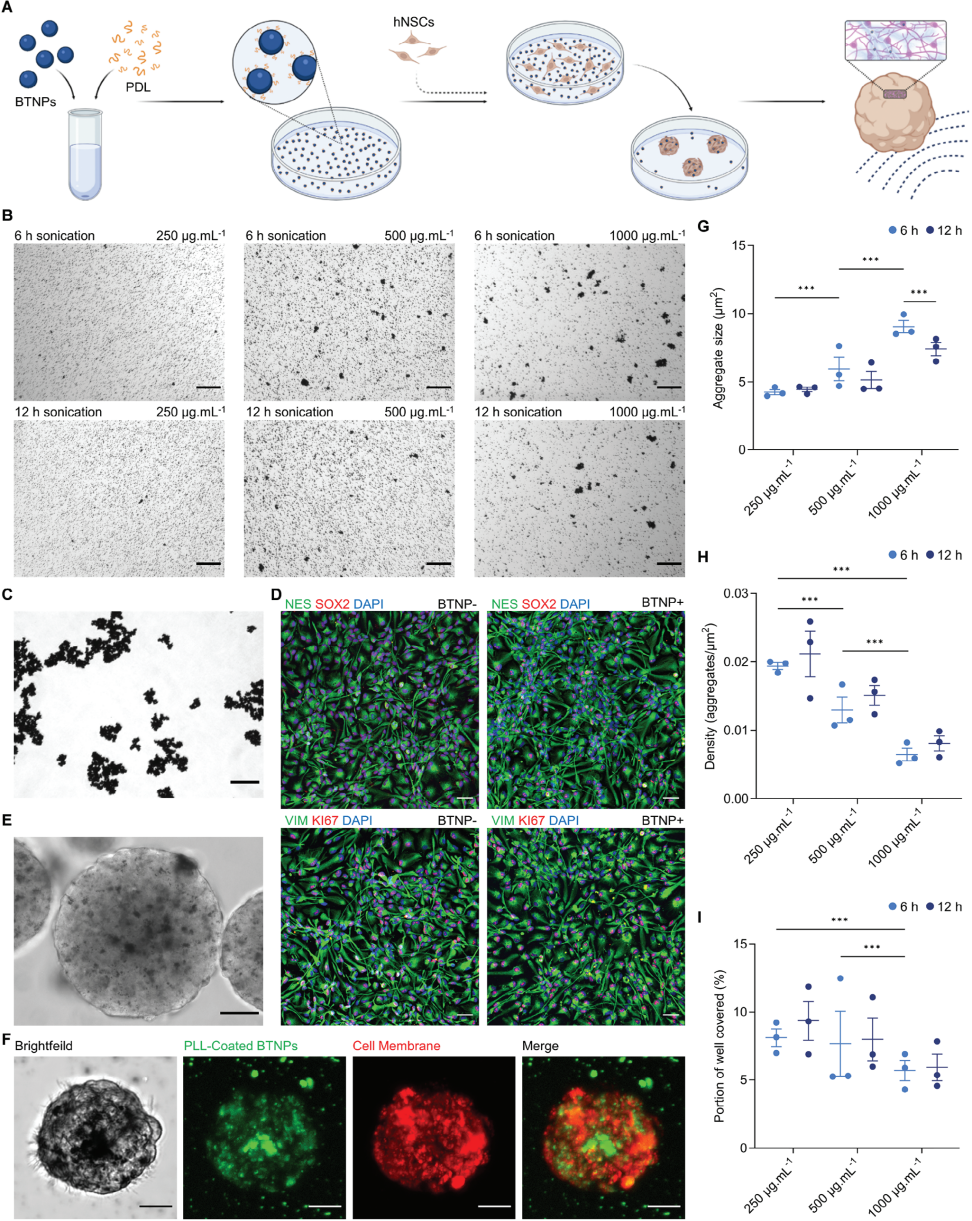
PDL coating of BTNPs enhanced their dispersibility and uptake to form BTNP‐loaded neural spheroids. A) Experimental design: BTNPs were dispersed in PDL via sonication and then used to coat wells of tissue culture plates. hNSCs were then seeded into the PDL‐BTNP‐coated wells to form BTNP‐loaded neural spheroids and then exposed to ultrasound to induce local electric fields within the spheroids. B) Brightfield microscopy of different concentrations (250, 500, and 1000 µg mL^−1^) of PDL‐BTNPs coating wells following sonication for 6 or 12 h. Scale bar: 75 µm. C) Brightfield microscopy of an uncoated BTNP dispersion 500 µg mL^−1^. Scale bar: 75 µm. D) hNSC cultures with and without incorporated BTNPs following 5 days culture in proliferation media confirming stable expression of nestin (NES), SOX2, vimentin (VIM), and KI67. Scale bar: 50 µm. E) Brightfield microscopy of a detached neural spheroid (≈230 µm diameter) following culture on 500 µg mL^−1^ PDL‐BTNPs with BTNP loading of the spheroid is clearly evident. Scale bar: 50 µm. F) Confocal fluorescence microscopy z‐stack of a neural spheroid (≈75 µm diameter) showing PLL‐BTNPs relative to hNSC plasma membranes (PLL‐BTNPs: green, plasma membrane: red). BTNPs were coated with PLL‐FITC, and cell membranes (including intracellular membranes, liposomes, and lipoproteins) were labeled with VybrantTM CM‐DiI. Scale bar: 20 µm. See also Video S1 (Supporting Information). G) Average aggregate size (µm^2^), H) average aggregate density (count/area (µm^2^)), and I) area covered (%) by PDL‐BTNPs in 6 and 12 h sonication samples. G–I) Mean values of technical replicates (*n* = 3) are shown together with the mean. Error bars denote SEM. Differences between the means were determined using a two‐way ANOVA followed by Bonferroni's posthoc multiple comparisons where *p* < 0.001 (^***^).

Piezoelectric nanomaterials are mechanotransducers capable of converting remotely generated mechanical stress into local endogenous electric fields for targeted stimulation. This energy transduction, known as the direct piezoelectric effect, originates from structural distortions of the crystalline materials, causing a loss of the center symmetry inducing electrical polarisation under mechanical stress.^[^ ^11^ ^]^ Mechanical deformation of piezoelectric nanomaterials via propagating ultrasound waves with frequencies of ≈20 kHz, permits ultrasound‐mediated piezoelectricity (USPZ), which is a promising neurostimulation modality, as well as antineoplastic and antibacterial.^[^ ^11^ ^]^ By enabling targeted, wireless, minimally invasive, localized electrical cues the approach overcomes the limitations of conventional electrical stimulation methods. It has been applied to modulate neural cell process outgrowth and branching,^[^ ^12^ ^]^ enhance neuronal cell differentiation and activation of calcium channels.^[^ ^13^ ^]^ and augment neural network activity.^[^ ^14^ ^]^ However, research to date has been limited to USPZ within 2D planer cell culture systems, rather than advanced 3D cellular systems. Whilst 2D cultures provide ease of handling and study, they fail to recapitulate the native spatial arrangement of neural cells and 3D microenvironment, omitting tissue‐relevant cellular behavior and dynamic and complex interaction with the surrounding extracellular milieu. 3D models of the human brain therefore aim to establish a more biologically and clinically relevant arrangement of cells and environment to better reflect the complexity and function of in vivo tissue. This includes a complex and dynamic neuronal circuitry, for enhanced understanding of neural tissue development and neuropathology.^[^ ^15^ ^]^ Here, we describe a new approach to in vitro neural cell and tissue modeling with USPZ stimulation through the generation of 3D piezoelectric human neural spheroids and derivative tissues. Our method uses piezoelectric barium titanate nanoparticles (BTNPs) that are incorporated into 3D self‐organizing hNSC‐derived spheroids, for subsequent nanoparticle activation by ultrasound (**Figure** **1**A). Nanoparticle activation enables 3D electrical stimulation throughout the spheroids for enhanced neuronal induction and function and augmented neural tissue formation and maturation. Resultant constructs can be used for extended tissue modeling, inclusive of modeling electroceuticals, as well as potential replacement tissues with or without stimulation.

## 2 Result

### 2.1 Preparation of Stable Barium Titanate Nanoparticle Dispersions

Ultrasonication enabled the preparation of stable suspensions of BTNPs in dispersant Poly‐D‐Lysine (PDL‐BTNPs) for subsequent experimentation. BTNP suspensions comprised dispersed individual particles and small aggregates of particles. In contrast, uncoated BTNPs formed unstable and large aggregate macro‐clusters (Figure 1B,C). Studies of time of sonication (6 or 12 h) for BTNP coating with PDL and subsequent dilution in PBS demonstrated no effect on aggregate size (*F*(2, 208) = 5.665, *p *> 0.01, partial η^2^ = 0.052), aggregate density, (*F*(2208), = 0.114, *p* > 0.001, partial η^2^ = 0.001), and the portion of the well covered (*F*(1, 208) = 17.027, *p* > 0.05, partial η^2^ = 0.011). The concentration of PDL‐BTNPs significantly impacted particle dispersion, with lower concentrations associated with reduced aggregate size (*p *< 0.001), increased particle density (*p* < 0.001), and greater and more uniform coverage of the wells (*p* < 0.001) (Figure 1G–I).

### 2.2 Formation of Barium Titanate Nanoparticle‐Laden Spheroids

**Figure 2:**
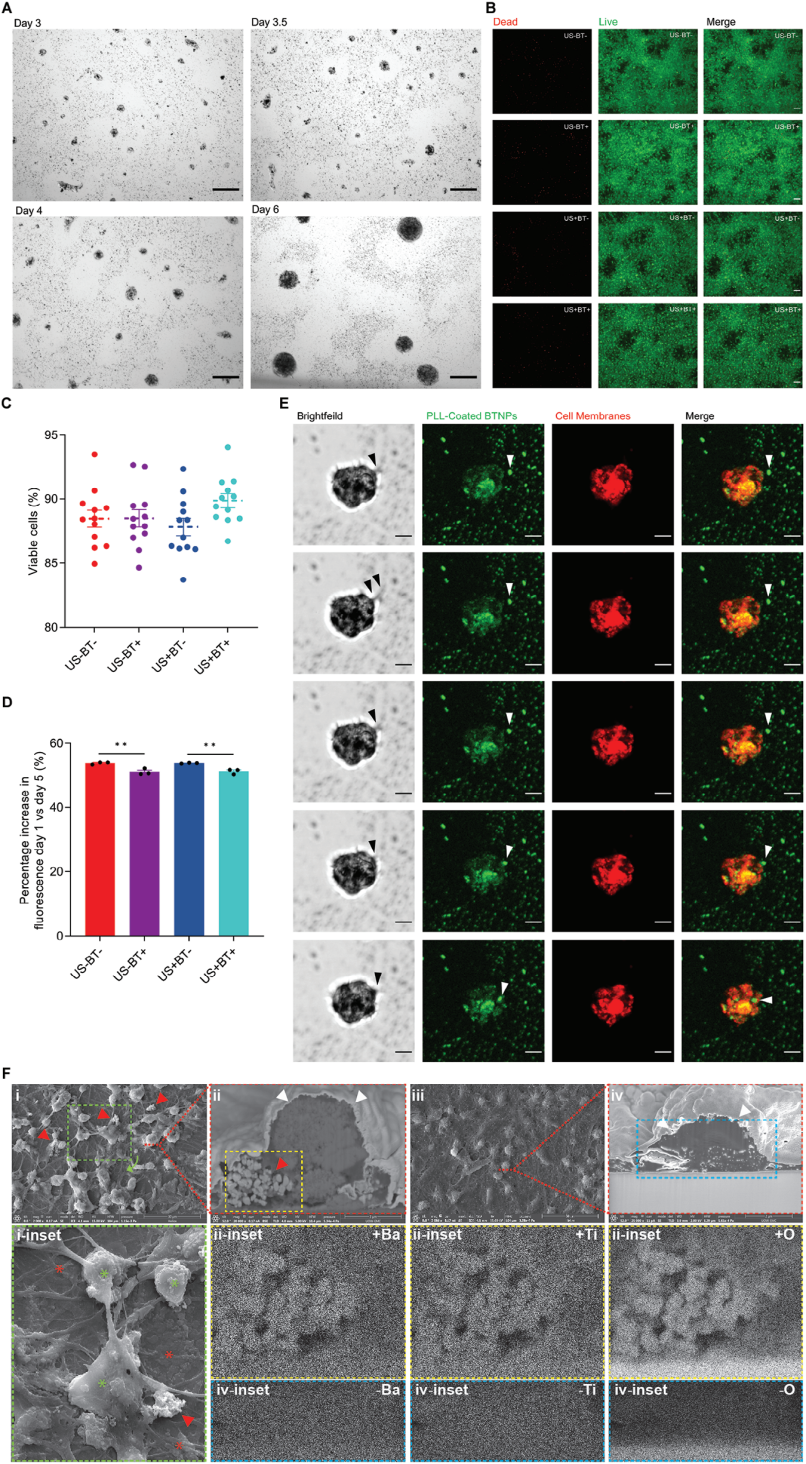
PDL‐BTNP uptake by hNSC spheroids did not affect cell viability. A) hNSC spheroid formation with incorporated BTNPs and associated BTNP clearance “halos” on 500 µg mL^−1^ PDL‐BTNP substrate. Scale bars: 75 µm (day 3, day 3.5, day 4) and 150 µm (day 6). B) Representative microscopy images of live (Calcein AM, green) and dead (PI, red) cells. Scale bars: 300 µm. C) Quantitative analyses of live and dead cells. There was no significant interaction between PDL‐BTNPs and US that affected the percentage of live cells as assessed by two‐way ANOVA (*F*(1,44) = 2.475, *p* < 0.05, partial η^2^ = 0.053). D) Quantitative analysis of cell proliferation from day 1 to day 5 following differentiation. There was no significant interaction between PDL‐BTNPs and US that affected cell proliferation (*F*(1,8) = 0.054, *p *> 0.05, partial η^2^ = 0.007), however, the presence of PDL‐BTNP reduced proliferation, as assessed by two‐way ANOVA (*F*(1,8) = 49.850, *p *< 0.001 partial η^2^ = 0.862). E) Confocal microscopy time‐lapse images of BTNP uptake by hNSCs of a neural spheroid (≈50 µm diameter) after 4 days of incubation (BTNPs: green; cell membranes: red). BTNPs were coated with PLL‐FITC, and cell membranes (including intracellular membranes, liposomes, and lipoproteins) were labeled with VybrantTM CM‐DiI. Black arrowheads indicate filopodia movement, with white arrowheads indicating BTNP displacement and cellular internalization. Scale bar: 20 µm. F) SEM images of immobilized neural spheroids with (F‐i, ii) and without (F‐iii, iv) BTNPs, confirming inter‐ (F‐i) and intra‐ (F‐ii) cellular uptake/localization of individual and aggregates of BTNPs (red arrowheads), with the diameter of BTNPs being ≈200 nm, and EDS characterization showing the nanoparticles consist of BA (Fii‐inset; ‐Ba = Fiv‐inset negative control) and Ti (Fii‐inset; ─Ti = Fiv‐inset negative control), and O (Fii‐inset; ─O = Fiv‐inset negative control) elements, with the ratio of Ba:Ti:O ≈1:1:3, in accordance with the chemical formula of BaTiO3. White arrowheads indicate the cell membranes of neurons in the region of the soma (F‐ii, iv) and Fi‐inset shows neuronal cell (green stars) and glial cell (flattened, red stars) morphology, with interconnecting dendrites and axonal projections emanating from neuronal cell bodies. See also Videos S2 and S3 (Supporting Information) for time‐lapse live‐cell imaging.

hNSCs seeded onto the surface of wells of tissue culture plates coated with PDL‐BTNPs proliferated and aggregated to form loosely attached spheroids (Figure 1E). Filopodia‐like extensions emanated from cells of developing spheroids and sequestered adjacent BTNPs. This resulted in the clearance of BTNPs from the areas surrounding spheroids and the incorporation of BTNPs within spheroids ≈3 days after cell seeding (**Figure** **2**A). Immunophenotyping of spheroids with and without PDL‐BTNPs demonstrated expression of neural progenitor markers vimentin (VIM), nestin (NES), and SOX2, and the cellular proliferation marker KI67 (Figure 1D).

Quantification of neural spheroid attachment to the culture plates following formation revealed statistically significantly increased detachment with the addition of PDL‐BTNPs (83.875 ± 22.375 µm) compared to the negative control condition (64.812 ± 19.759 µm) (t(30) = 2.554, *p* < 0.05). In addition, the mean diameter of spheroids with incorporated PDL‐BTNPs was greater (74.663 ± 19.988 µm) compared to negative control spheroids (68.201 ± 18.275 µm) (U = 507 395, z = −8.075, *p* < 0.001). Furthermore, increased concentrations of PDL‐BTNPs were associated with an increased number of spheroids formed. The number of detached spheroids with 250, 500, and 1000 µg mL^−1^ PDL‐BTNPs were 8.833 ± 7.134, 103.000 ± 15.663, and 203.333 ± 17.062, respectively. One‐way analysis of variance (ANOVA) indicated a significant main effect of PDL‐BTNP concentration (*F*(2,30) = 0.010, *p *< 0.001, partial ƞ^2^ = 0.440). Collectively, these results indicate that 500 µg mL^−1^ PDL‐BTNP is optimal for forming BTNP‐loaded neural spheroids, with minimal nanoparticle aggregation but high nanoparticle concentration for effective piezoelectric stimulation.

BTNP internalization within hNSCs was characterized by live‐cell confocal microscopy and focussed ion beam scanning electron microscopy (FIB‐SEM). For live‐cell imaging, BTNPs were coated with Fluoresceine (FITC)‐labelled Poly‐L‐Lysine (PLL), the naturally occurring enantiomer of PDL with the same charge‐related properties and cellular interactions. BTNP coating with PLL‐FITC revealed the internalization of BTNP clusters within the cells of the hNSC spheroids. BTNPs appeared as opaque regions within the neural spheroids observed by transmitted light and colocalized with FITC fluorescence, as detected by confocal laser scanning microscopy (Figure 1F; Video S1, Supporting Information). Filopodia/lamellopodia protrusions of several micrometers to tens of micrometers in length extended from neural spheroids and displayed distinctive sweeping, clearance, and uptake of surrounding BTNPs (Figure 2E; Videos S2 and S3, Supporting Information). Remarkably, live‐cell imaging and SEM confirmed BTNPs were incorporated into the spheroids both inter‐ and intra‐cellularly, with the process of nanoparticle uptake occurring ≈180 sec after initial filopodium contact with BTNPs (Videos S2 and S3, Supporting Information; Figure 2F).

### 2.3 Cell Viability and Proliferation Following Barium Titanate Nanoparticle Uptake by Spheroids and Activation by Ultrasound

Cell viability assays indicated no significant cell death of spheroids loaded with PDL‐BTNPs with or without ultrasound exposure (US+BT+ and US‐BT+, respectively), or exposed to ultrasound without PDL‐BTNPs (US+BT‐) compared to control samples without ultrasound exposure or PDL‐BNTPs (US‐BT‐). As such, statistical analysis of cell viability revealed no significant interaction between PDL‐BTNPs and ultrasound (F(1,44) = 2.475, *p* > 0.05, partial η^2^ = 0.053). Taken together, these results indicate that PDL‐BTNPs and exposure to ultrasound with or without PDL‐BTNPs for 5 days do not significantly affect the cell viability of spheroids (Figure 2B,C).

Cell proliferation assays indicated that all conditions (US‐BT+, US+BT‐, US+BT+) were associated with an increase in cell proliferation (Figure 2D). Statistical analysis of cell proliferation revealed no significant interaction between PDL‐BTNPs and ultrasound (*F*(1,8) = 0.054, *p* > 0.05, partial η^2^ = 0.007). However, analysis of the simple main effects revealed that the presence of PDL‐BTNPs within spheroids affected cell proliferation (*F*(1,8) = 49.850, *p* < 0.001 partial η^2^ = 0.862). Specifically, the mean percentage increase in cell proliferation of spheroids with PDL‐BTNPs was significantly lower compared to spheroids without PDL‐BTNPs (51.155 ± 0.930 and 53.814 ± 0.368, respectively; 95% CI, 1.470 to 3.848%, *p* < 0.01). Similarly, ultrasound treatment of spheroids with PDL‐BTNPs resulted in a reduced percentage increase in cell proliferation compared to spheroids without PDL‐BTNPs (51.314 ± 0.126 and 53.804 ± 0.126, respectively; 95% CI, 1.030 to 3.678%, *p *< 0.01). To summarise, the proliferative capacity of differentiating hNSCs of spheroids increased during culture for all test conditions. However, whilst the percentage increase was significantly less for cells cultured with PDL‐BTNPs, USPZ stimulation did not impact cell proliferation.

### 2.4 Immunophenotyping Neural Tissue Induction, Neuronal Networking and Maturation Following Ultrasound‐Mediated Piezoelectric Stimulation of Spheroids

**Figure 3:**
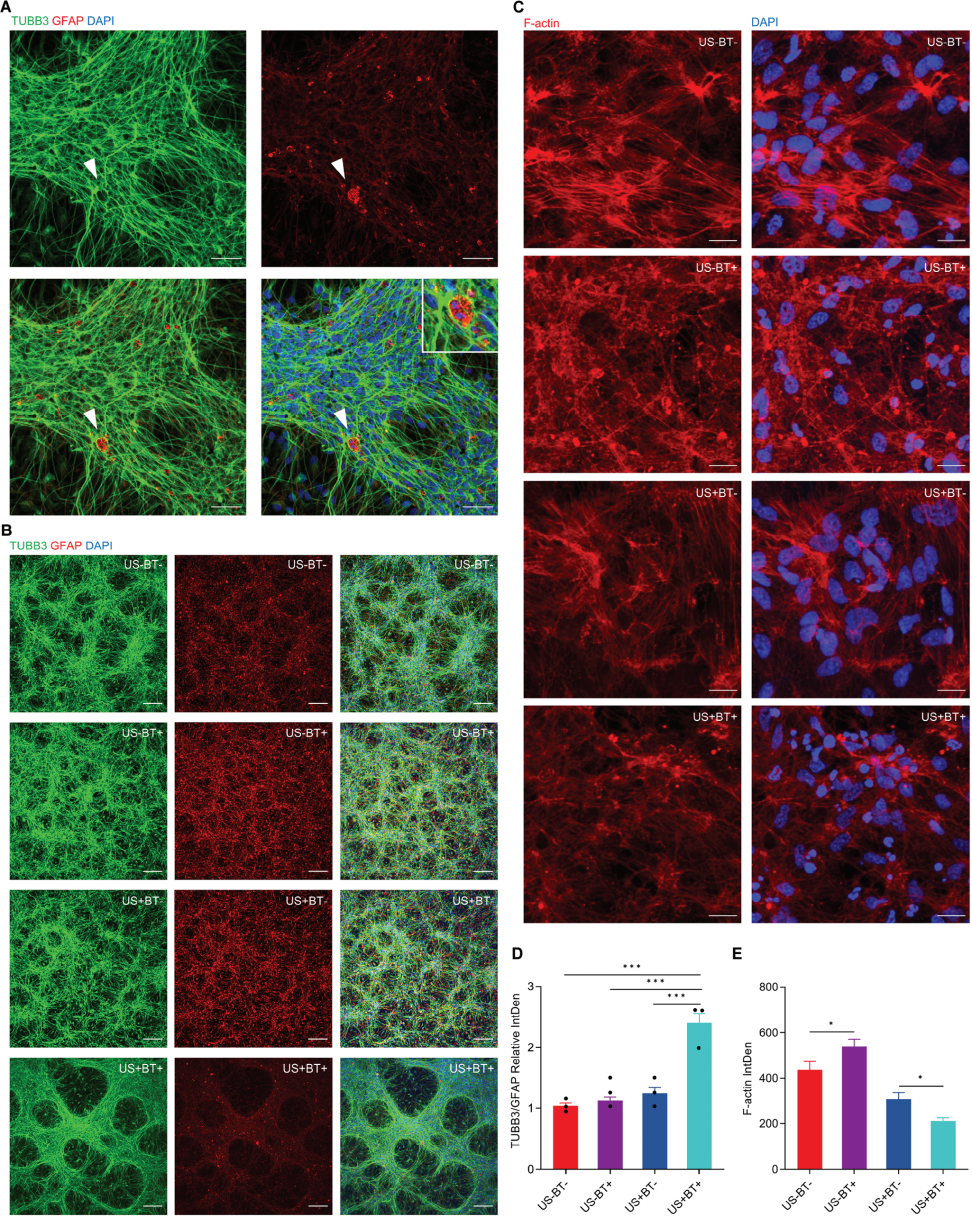
USPZ stimulation enhanced neuronal induction from neural spheroids and induced F‐actin reorganization of derivative tissues. A) Immunophenotyping following USPZ stimulation indicated induction of early neuronal (TUBB3 expressing) cells and neuroglia (GFAP expressing). White arrowheads highlight axon projections extending between cell clusters with terminal swellings/boutons abutting glia (inset). Scale bar: 50 µm. B) Expression of TUBB3 and GFAP of control (US‐BT‐, US‐BT+, US+BT‐) and USPZ stimulated (US+BT+) samples, with stimulated tissues showing a marked 3D “honeycomb”‐like tissue‐structure. Scale bars:150 µm. C) Confocal z‐projection of F‐actin labeling (phalloidin labeling, green) with and without incorporated PDL‐BTNPs, with and without US stimulation. Scale bar: 20 µm. D) Quantitative assessment of immunocytochemistry showing the ratio of TUBB3 to GFAP expression (Integrated Density; IntDen). USPZ stimulation induced higher TUBB3 expressing neurons, with reduced induction of GFAP expressing glial cells compared to unstimulated samples (*F*(1695) = 29.606, *p *< 0.001 partial η^2^ = 0.041; Two‐way ANOVA with Bonferroni post‐hoc test; ^***^ *p* < 0.001). Mean values for each independent replicate (*n *= 3) are shown together with the overall mean. Error bars denote SEM. E) Quantitative assessment of F‐actin immunocytochemistry (Integrated density; IntDen). Error bars denote SEM, (*n *= 1). Notably, USPZ stimulation associated with reduced F‐actin IntDen (*F*(2,84) = 12.638, *p *< 0.001, partial η^2^ = 0.231; Two‐way ANOVA with Bonferroni post‐hoc test; ^*^ *p* < 0.05).

**Figure 4:**
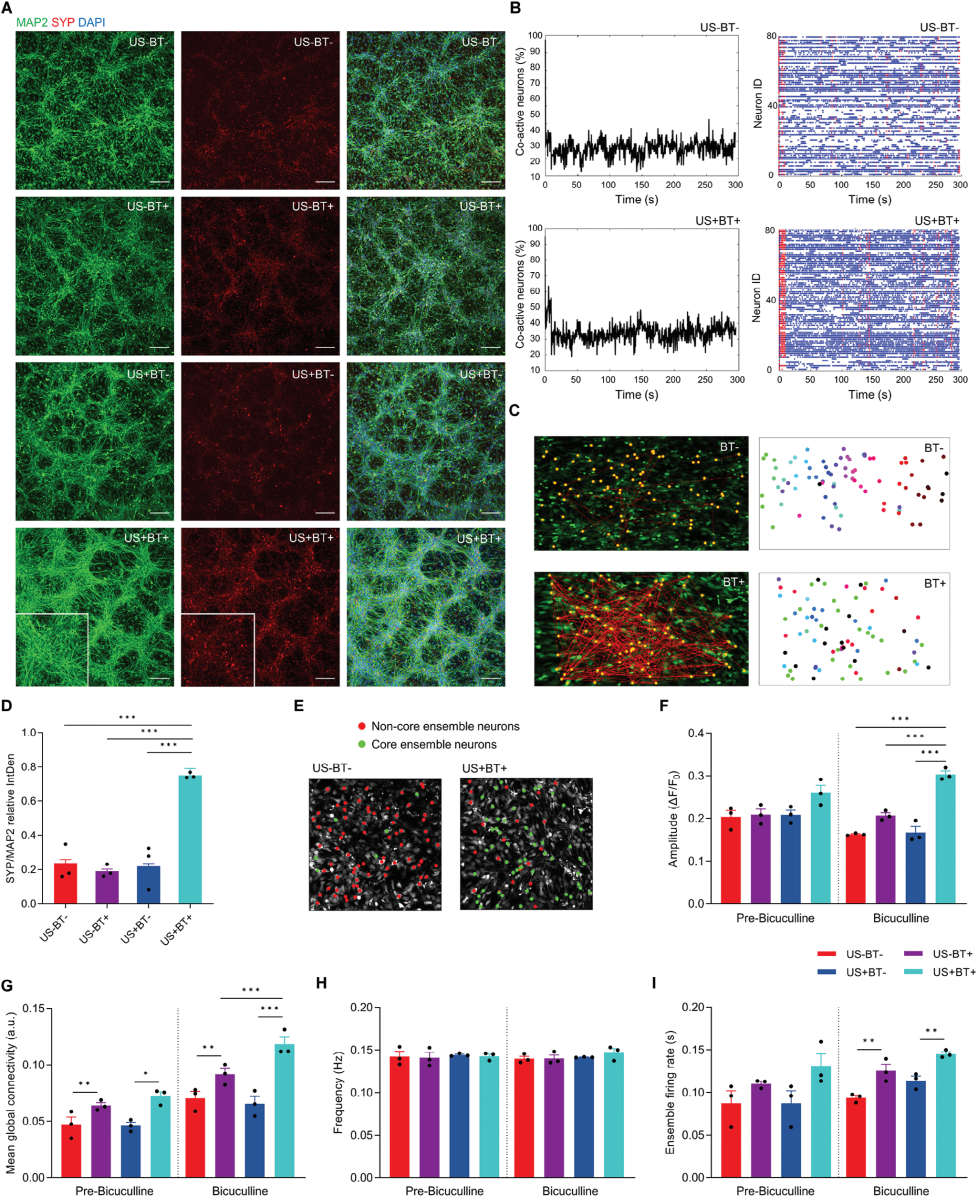
USPZ stimulation enhanced neuronal maturation, synaptic density, neural firing amplitude and rate, and connectivity. A) Immunophenotyping control (US‐BT‐, US‐BT+, US+BT‐) and USPZ stimulated (US+BT+) tissues showing mature neuronal MAP2 (green; inset: uniform labeling of cell bodies and neurites) and presynaptic glycoprotein SYP (red; inset: puncta decorating cell bodies and neurites consistent with synaptic localization), with stimulated tissues again showing a marked 3D “honeycomb”‐like tissue‐structure. Scale bar: 150 µm. B) Analysis of live‐cell calcium flux for unstimulated (US‐BT‐) and USPZ stimulated tissues, showing the percentage of coactive neurons over time and a corresponding raster plot of spontaneous activity using threshold spike data. Each horizontal row (blue) indicates the activity of a single neuron, with vertical lines (red) denoting statistically significant coactive neurons. C) Topographic maps of live‐cell calcium flux demonstrating the effect of incorporated BTNPs on functional connectivity of neurons (yellow dots represent firing neurons, red dashed lines represent functional connections) and visualization of corresponding neural maps, with like‐colors indicating connected neurons. D) Quantitative assessment of SYP and MAP2 immunochemistry, with indices of neuronal maturation and networking derived from ratios of SYP to MAP2 expression levels (Integrated Density; IntDen) indicating more mature neuronal tissue for USPZ stimulated compared to unstimulated tissues (*F*(1691) = 157.619, *p* < 0.001 partial η^2^ = 0.196; Two‐way ANOVA with Bonferroni posthoc test; ^***^ *p* < 0.001). E) Representative image indicating core ensemble neurons (green) and neurons not a part of the core ensemble (red). F) Quantitative analysis of live‐cell calcium flux indicated a statistically significant increase in the mean spike amplitude (ΔF/F_0_) for USPZ stimulated (US+BT+; 0.303 ± 0.010 ΔF/F_0_) compared to unstimulated controls (US‐BT+:0.207 ± 0.020 ΔF/F_0_, *p* < 0.001; US‐BT‐:(0.163 ± 0.011 ΔF/F_0_, *p* < 0.001; US+BT‐: 0.167 ± 0.024 F/F_0_,(*p* < 0.001). G) Quantitative analysis of live‐cell calcium flux indicated mean global connectivity of neurons was increased for USPZ stimulated tissues treated with GABA_A_ antagonist bicuculline (*F*(1,11) = 6.881, *p* < .01, partial η^2^ = 0.5; Two‐way ANOVA with Bonferroni posthoc test; ^*^ *p* < 0.05, ^**^ *p* < 0.01, ^***^ *p* < 0.001; a.u., arbitrary unit. H) Quantitative analysis of live‐cell calcium flux indicated no effect of USPZ stimulation on spontaneous firing frequency without (*F*(1,12) = 0.18, *p* > 0.05, partial η^2^ = 0.74) or with (*F*(1,12) = 0.16, *p* > 0.05, partial η^2^ = 0.95) bicuculline treatment. I) Pairwise comparisons revealed USPZ stimulation (0.145 ± 0.004 s) enhanced ensemble firing frequency compared to unstimulated controls (US‐BT+: 0.114 ± 0.009 s, *p* < 0.01). US‐BT+: 0.125 ± 0.013 s, *p* < 0.01) had a higher ensemble firing frequency than US‐BT‐ (0.094 ± 0.005 s, *p* < 0.01). D,F–I) The mean values of each independent replicate (*n *= 3) are shown together with the overall mean. Errors bars denote SEM.

Immunophenotyping revealed enhanced hNSC differentiation and ensuing neural tissue induction and maturation of USPZ‐stimulated spheroids. Tissues developed from spheroids that merged during differentiation and stimulation to form mats (1.7 cm^2^) of densely packed, interwoven, and interconnected neuronal class III beta‐tubulin (TUBB3) (**Figure** **3**A) and microtubule associated protein 2 (MAP2) (**Figure** **4**A) expressing neurons (characterized by elongated radially projecting neurites), and dispersed glial fibrillary acidic protien (GFAP) expressing glia (Figure 3A). The arrangement of the neurons resulted in 3D “honeycomb”‐like tissue‐structures, less apparent for control tissues (Figures 3A and 4A). In addition to neuronal networking, long axonal‐like projections were observed to abut neuroglia as terminal swellings/boutons (Figure 3A).

Quantitative analyses of TUBB3 and GFAP immunocytochemistry supported qualitative assessments, with USPZ stimulation inducing hNSCs to predominately TUBB3 expressing neurons, with reduced induction of GFAP expressing glial cells (*F*(1695) = 29.606, *p* < 0.001 partial η^2^ = 0.041) compared to unstimulated samples (Figure 3B,D). Moreover, colocalization of synaptophysin (SYP) with the high‐density mature MAP2 expressing neurons indicated augmented neuronal networking and maturation of USPZ stimulated tissues compared to control tissues (Figure 4A). Quantitative analysis revealed an enhanced ratio of SYP to MAP2 (*F*(1691) = 157.619, *p* < 0.001 partial η^2^ = 0.196) corroborating enhanced neuronal maturation compared to unstimulated controls (Figure 4A,D).

### 2.5 F‐Actin Reorganisation Following Barium Titanate Nanoparticle Uptake and Ultrasound‐Mediated Piezoelectric Stimulation of Spheroids

Labeling of neural tissues with fluorophore‐conjugated phalloidin revealed substantial F‐actin reorganization and enhanced expression in response to uptake of PDL‐BTNPs (US‐BT+: 533.794 ± 276.660 IntDen; versus US‐BT‐: 438.478 ± 272.980, mean difference = 95.316 IntDen, 95% CI, 9.938 to 200.570 IntDen, *p *< 0.05; Figure 3C,E). Moreover, the uptake of PDL‐BTNPs was associated with the disassembly of stress fibers to form short and branched actin networks, with bright fluorescent puncta. In contrast, tissues without nanoparticles (US‐BT‐) comprised cells with arrays of actin filaments forming parallel and elongated stress fibers that fanned across the cytosol. Furthermore, USPZ of tissues with incorporated PDL‐BTNPs (US+BT+; 211.330 ± 104.827 IntDen) caused both disassembled F‐actin and reduced fluorescence intensity compared to US+BT‐ control tissues (mean difference = 97.334 IntDen, 95% CI, 11.883 to 206.572 IntDen, *p* < 0.05). Upon closer examination, conformational alterations of F‐actin or stress fiber disassembly after USPZ appeared even more progressed compared to ultrasound‐only (US+BT‐) spheroids, with even greater observed anisotropy of actin networks and shortening of filaments (Figure 3C,E).

### 2.6 Neuronal Networking and Function Following Ultrasound‐Mediated Piezoelectric Stimulation of Spheroids

Assessment of neuronal networking and function supported recurrent spontaneous fluctuations in intracellular calcium concentrations of both unstimulated and USPZ‐stimulated tissues (Figure 4B). There was no significant interaction between ultrasound and BNTPs that affected the spontaneous spike amplitude of neurons (*F*(1,11) = 2.600, *p* > 0.05, partial η^2^ = 0.290). However, when combined with disinhibition of cells by gamma‐aminobutyric acid receptor‐A (GABA_A_) antagonist bicuculline, USPZ stimulation induced an increased spike amplitude of calcium flux (*F*(1,11) = 27.485, *p* < .01, partial η^2^ = 0.750). Interestingly, the mean amplitude for USPZ stimulated tissues (0.303 ± 0.010 ΔF/F_0_) was statistically significantly higher compared to unstimulated tissues (US‐BT+: 0.207 ± 0.020 ΔF/F_0_, *p* < 0.001; US‐BT‐: 0.163 ± 0.011 ΔF/F_0_, *p* < 0.001; US+BT‐: 0.167 ± 0.024 ΔF/F_0_, *p* < 0.001; Figure 4F). Further, USPZ‐stimulated tissues treated with bicuculline exhibited enhanced global connectivity of neurons compared to more localized connections in unstimulated tissues. The functional connectivity of neurons (expressed as arbitrary units (a.u.)) was defined as a significant temporal correspondence of calcium events between two or more neurons (*p* < 0.05). Following the addition of bicuculine, there was a significant positive interaction between ultrasound stimulation and BTNPs on the mean global connectivity within neurons (*F*(1,11) = 6.881, *p *< .01, partial η^2^ = 0.5). The mean global connectivity for USPZ stimulated neural tissues (0.118 ± 0.011 a.u.) was statistically significantly higher than unstimulated controls US‐BT+: 0.092 ± 0.009 a.u., *p* < 0.05; US‐BT‐:0.070 ± 0.010 a.u., *p* < 0.001; US+BT‐: 0.066 ± 0.011 a.u., *p* < 0.05; Figure 4C,F).

The mean spike frequency of intracellular calcium flux in individually firing neurons was not effected by USPZ stimulation, consistent with the mean frequency (Hz) of both spontaneously firing (*F*(1,12) = 0.18, *p* > 0.05, partial η^2^ = 0.74) and bicuculine induced firing (*F*(1,12) = 0.16, *p* > 0.05, partial η^2^ = 0.95 (Figure 4H). However, it is interesting to note that once individually firing neurons were organized into functional ensembles the frequency of firing varied between conditions. Network ensembles were defined as groups of neurons in which a statistically significant number of cells were co‐active for a time frame. Whilst there was no significant effect of USPZ stimulation on the ensemble firing rate of bicuculine treated tissues (*F*(1,12) = 0.003, *p* > 0.05, partial η^2^ = 0.000), pairwise comparisons demonstrated USPZ stimulation (0.145 ± 0.004 s) induced more frequent ensemble firing by 0.031 s (95% CI 0.015 to 0.047 s, *p* < 0.01) compared to tissues without BTNPs exposed to ultrasound (US+BT‐: 0.114 ± 0.009 s). Similarly, tissues with BTNPs not exposed to ultrasound (US‐BT+: 0.125 ± 0.013 s) exhibited a higher ensemble firing frequency of 0.032 s (95% CI.15 to.048 s, *p* < 0.01) compared to tissues without BTNPs and ultrasound exposure (US‐BT‐: 0.094 ± 0.005 s) (Figure 4E,I). Furthermore, individual neurons firing in one ensemble were also active in other ensembles with a different set of neurons, suggesting that individual neurons flexibly shift from ensemble to ensemble.

## 3 Discussion

The use of exogenous electric fields to modulate stem cell fate is gaining increasing attention. Considering that electrical activity plays a vital role in the early development of the nervous system, its increasing use for stem cell‐derived neural cell induction and modulation especially, is not surprising. Interest is also driven by the need for better approaches to neural tissue engineering for in vitro modeling and regenerative medicine, as well as electroceuticals in neurology. More contemporary methods to deliver electrical stimulation to cells include wireless systems such as ultrasound‐mediated piezoelectricity.^[^ ^17^ ^]^ Despite its promise, USPZ application has been mostly limited to non‐human (more specifically rodent) and planar cell culture and is yet to be applied for stem cell culture and differentiation. Here we describe the application of USPZ stimulation for human neural tissue induction and maturation from hNSCs, including a novel approach to incorporating piezoelectric nanoparticles in 3D hNSC‐derived spheroids during their formation in vitro. Our piezoelectric 3D human neural tissue models, therefore, represent the first example of USPZ enhancement of human neuronal induction from stem cells, maturation (including augmented neuritogenesis and synaptogenesis), function (enhanced intracellular calcium signaling), and drug responsivity (exemplified by bicuculline treatment). Furthermore, our method involves novel direct tissue loading with piezoelectric particles and their wireless activation with ultrasound. Conceding the inherent difficulty with precisely measuring the electrical output of piezoelectric nanoparticles, electro‐elastic mathematical modeling predicts an output voltage of ≈0.5 mV by the BTNPs presently employed in response to a US intensity of 0.8 W cm^−2^.^[^ ^12c^ ^]^ This together with the phenotypic and functional cell effects of USPZ stimulation, supports BTNP‐assisted USPZ stimulation in our 3D tissue models. It is therefore anticipated that the platform described will facilitate advanced tissue engineering and in vitro modeling of human neural (and potentially non‐neural) tissues, with modeling including tissue development and pathology, and applicable to pre‐clinical testing and prototyping both electroceuticals and pharmaceuticals.

BTNPs possess distinctive piezoelectric and dielectric properties, rendering them appealing for USPZ and tissue engineering applications.^[^ ^11^, ^12^, ^18^ ^]^ However, the overall negative charge and hydrophobic nature of BTNPs results in particle aggregation in aqueous solutions, thus impeding the production of a homogenous dispersion.^[^ ^19^ ^]^ We have used the synthetic poly(amino acid) PDL to prevent aggregation and potential cytotoxicity from Ba^2+^ leaching,^[^ ^20^ ^]^ as well as enhance particle hydrophilicity by adsorbing onto the cationic BTNP surface through electrostatic interaction of the positively charged NH_2_ group. PDL is one of two enantiomers of Poly‐Lysine; the other being PLL. While the PDL precursor amino acid is an artificial product, the PLL precursor occurs naturally. The former is therefore resistant to enzymatic degradation and therefore our preferred choice for coating BTNPs for USPZ stimulation of 3D neural tissues. Previously established sonication protocol for BTNP surface coating with a polyelectrolyte species.^[^ ^21^ ^]^ was presently modified from 12 h of sonication to 6 h of sonication with negligible impact on particle aggregation. Uncoated BTNPs were not incorporated into neural spheroids demonstrating the need for polymeric wrapping/coating of BTNPs. The coating provides positively charged hydrophilic amino groups on the BTNP exterior, altering the surface charge and polarity, for biocompatibility.^[^ ^21^, ^22^ ^]^ Whilst there is discord in the literature regarding surface charge and cellular compatibility, positively charged molecules are thought to be more cell‐compatible than negatively charged particles due to their electrostatic interaction with negatively charged cell membranes.^[^ ^22^, ^23^ ^]^ Here we report the total number of spheroids and the spheroid diameter increased with increasing concentrations of PDL‐BTNPs. Additionally, cell culture with PDL‐BTNPs increased neural spheroid formation relative to “free” PDL not complexed with BTNPs. Taken together, these results indicate that polymeric BTNP coating enables stable, homogenous dispersions of BTNPs and augments uptake and incorporation by neural spheroids during formation. Furthermore, the formed constructs initially develop into 3D BTNP‐laden microtissues comprising multipotent, self‐renewing hNSCs that can be differentiated into neuronal cells and supporting neuroglia.

BTNP uptake filopodia/lamellopodia protrusions of the neural spheroids is consistent with the known functions of filopodia involving adhesive contact with the extracellular matrix, pathogens, and adjacent cells or particles to exert pulling forces for engulfment.^[^ ^24^ ^]^ Macrophage filopodia have demonstrated capture of latex beads,^[^ ^25^ ^]^ carboxylated beads,^[^ ^24b^ ^]^ as well as beads covered with bacterial surface proteins.^[^ ^26^ ^]^ To the best of our knowledge, this is the first study demonstrating hNSC cellular capture of PDL‐BTNPs through a filopodium‐mediated mechanism. Filopodia‐mediated particle uptake can occur through three well‐known mechanisms; particle sweeping, filopodia retraction, and particle capture on the distal portion of the filopodial shaft with succeeding particle surfing toward the cell body.^[^ ^27^ ^]^ Live‐cell imaging revealed that particle sweeping and filopodia retraction were the primary mechanisms for PDL‐BTNP capture and engulfment. The observed intracellular uptake of nanoparticles occurs via endocytosis; either phagocytosis of particles larger than 500 nm, or pinocytosis of smaller particles.^[^ ^28^ ^]^ For the present study, individual BTNPs with a diameter of ≈200 nm or small macro clusters of particles were likely pinocytosed, whereas larger particle aggregates were likely phagocytosed. Key to the process are cytoskeletal microfilaments composed of actin polymers concentrated at the periphery of the cell. As such, actin is responsible for forming cytoplasmic filopodia and lamellipodia and providing resting tension and mechanical support for the cell body.^[^ ^29^ ^]^ The plasma membrane is functionally integrated with the underlying actin‐based cytoskeleton; thus, it is anticipated that the cellular internalization of PDL‐BTNPs requires reorganization of the cytoskeleton, affirming the filopodia/lamellopodia‐mediated endocytosis of PDL‐BTNPs. Our findings concur with reports of disruption of F‐actin following nanoparticle endocytosis,^[^ ^29^, ^30^ ^]^ whilst being the first account of PDL‐BTNP uptake by cells via filopodia‐mediated sweeping and retraction mechanisms with cytoskeletal reorganisation. Also notable were the conformational alterations of F‐actin or stress fiber disassembly after USPZ stimulation, with even greater observed anisotropy of actin networks and shortening of filaments compared to ultrasound treatment alone indicative of a potential synergistic action between electrical and mechanical cues on cellular dynamics. This effect of electrical stimulation on actin cytoskeleton reorganization is consistent with the previously reported effect of electrical stimulation on the mechanical characteristics of the cell plasma membrane, above mentioned plasma membrane and cell cytoskeleton interaction, and the purported influence of electrical stimulation on cell homeostasis (e.g., endo‐ and exocytosis, cell adhesion, motility, and related cell signaling) via membrane‐cytoskeleton coupling.^[^ ^31^ ^]^

USPZ stimulation did not significantly alter cell viability or impede cell proliferation, corroborating previous reports on the biocompatibility of USPZ.^[^ ^12^, ^13^, ^32^ ^]^ Whilst the accumulation of reactive oxygen species (ROS) has previously been reported following the internalization of BTNPs in several aquatic microorganisms,^[^ ^33^ ^]^ we found no significant alteration in the percentage of viable cells succeeding endocytic incorporation of PDL‐BTNPs into hNSCs. Further, the coating of piezoelectric nanoparticles with PLL has previously been shown to contribute to cellular necrosis, with the presence of 20 µg mL^−1^ of PLL‐BTNPs leading to cell death of H9C4 cells, due to the presence of PLL.^[^ ^21^ ^]^ However, our results do not corroborate their findings, where we observed considerably lower levels of cell necrosis despite employing a 25‐fold increase in the concentration of PDL. Notwithstanding cell viability, tissues with incorporated PDL‐BTNPs, regardless of ultrasound exposure, exhibited a significant decrease in the percentage increase of proliferating cells during differentiation, potentially indicating that PDL‐BTNP loaded spheroids undergo enhanced neural differentiation and so contain terminally differentiated postmitotic cells relatively early during development.^[^ ^34^ ^]^

USPZ induced hNSCs to predominantly neurons, as demonstrated by enhanced fluorescence of TUBB3 and MAP2. The composition of neurons and neuroglial cells in the adult human brain varies between different brain regions, although until recently glial cells were thought to outnumber neurons in the human brain by 10:1. Applying newer counting methods has resulted in a revised glial:neural ratio of less than 1:1.^[^ ^35^ ^]^ Moreover, the observed tendency toward neuronal induction using USPZ corroborates previous reports of electrical stimulation causing enhanced neuronal induction.^[^ ^8^, ^36^ ^]^ Further, neural tissue exposure to USPZ demonstrated structural rearrangement of the cytoskeleton, which may be attributed to the altered biochemical environment during electrical stimulation. Increased cytosolic calcium concentration and enhanced cellular metabolic activity are linked to cytoskeleton disassembly.^[^ ^7c^ ^]^ These results concur with previous findings.^[^ ^37^ ^]^ that demonstrate conformational rearrangement of F‐actin filaments of human mesenchymal stem cells exposed to external electric fields. Remarkably, immunocytochemistry following USPZ revealed enhanced synaptogenesis with a higher number of presynaptic formations. Enhanced synaptophysin as a direct effect of exogenous electrical stimulation has been demonstrated, where deep brain stimulation was shown to partially restore synaptophysin expression following global ischemia in rat cortical neurons.^[^ ^38^ ^]^ Similarly, electrical stimulation via metal or printed conductive polymer pillar electrodes promotes synaptogenesis in primary rat cortical neurons.^[^ ^39^ ^]^ and hNSCs,^[^ ^36e^ ^]^ respectively. However, unlike the present study, these earlier reports utilized more conventional wired stimulation, whereas the current study employed a wireless approach to modulating hNSC differentiation for enhanced neuronal cell induction with increased synaptogenesis and augmented maturation.

Functional live‐cell calcium imaging of differentiated tissue revealed that USPZ enhanced intracellular calcium signaling, demonstrating the application of exogenous electric fields for heightened activation of calcium channels. Upon ultrasound stimulation, BTNPs induce a local electric field, which has been shown to enhance channel opening probability via generating an asymmetric shift of ions relative to the outer surface of the plasma membrane resulting in an alteration in cell polarity and a subsequent generation of electric potential and downstream molecular signaling. Additionally, the increased intracellular calcium, as a direct effect of USPZ, augments differentiation through two major molecular pathways; cAMP‐dependent and MAPK/ERK signaling cascades, providing insight into possible mechanisms of USPZ‐induced differentiation.^[^ ^13b^ ^]^

The bicuculline‐induced amplification of intracellular calcium spike and connectivity following USPZ reflects increased neural maturity, involving a GABAergic neuronal population.^[^ ^39^ ^]^ Maturing neural cultures undergo alterations in the balance of inhibition and excitation; in contrast to the adult brain, where GABA is the main inhibitory neurotransmitter, GABA exhibits excitatory functions in the developing brain.^[^ ^40^ ^]^ In control cultures without ultrasound or BTNPs, bicuculline treatment reduced excitatory activity and induced a low proportional increase in global connectivity, representing relatively immature tissue. The global connectivity and ensemble firing rate were further enhanced with USPZ stimulation of PDL‐BTNP‐loaded neural tissues. This is consistent with in vivo early development shifting from irregular isolated neural firing to complex networking propagating over larger areas. These results signify enhanced connectivity of USPZ‐stimulated neurons possibly attributable to changes in ionic currents, the balance between excitation and inhibition, and the maturity of surface receptors.^[^ ^41^ ^]^ It was identified that neuronal activity within ensembles influenced numerous other ensembles at different time points, indicating that individual neurons participate flexibly in various ensembles, potentially mimicking native neural tissue where neuron flexibility is a fundamental property of neural tissue function.^[^ ^42^ ^]^ Importantly, this data corroborates the live‐cell calcium imaging data indicating increased intracellular calcium as a direct response to USPZ. To summarise, USPZ increases the functional and structural maturation of 3D neural tissue cultures. These findings are important for engineering neural tissue more representative of in vivo mature tissue, disease modeling, and pharmacological assays for future translation.

## 4 Conclusion

Endogenous electric fields play an essential role in neural tissue development and homeostasis in vivo, from guiding embryonic brain development to maintenance and repair in adult tissue. Here, for the first time, we demonstrate the capability of USPZ for 3D human neuronal tissue engineering and remodeling. Notwithstanding the technical challenges with applying USPZ nanoparticle stimulation, including precise measurement and release of electricity, the need for a better understanding of the bioeffects, and challenges in clinical translation including regulatory requirements, for now, our preclinical/in vitro 3D piezoelectric neural tissue model endeavors to address the shortfalls of traditional 2D modeling by better replicating, for example, the in vivo neural microenvironment (inclusive of bioelectricity), cell organization and overall tissue function. The novel synthesis of 3D USPZ neural tissue could provide a workable platform for modeling mature neuronal phenotypes and functionalities, as well as a proof‐of‐concept for other neuronal and non‐neuronal tissue engineering and modeling.

## 5 Experimental Section

### Preparation and Assessment of Barium Titanate Nanoparticle Dispersions

Barium titanate nanopowder (200 nm, tetragonal, US Research Nanomaterials) was dispersed with PDL (Sigma Aldrich, P7280) by mixing equal quantities of the materials (1:1 w/w) followed by sonication (Branson Sonicator 3800) for 6–12 h) using an output power of 40 kHz. Samples were diluted 1:1, 1:2, and 1:4 in PBS and coated onto the surface of 24‐well tissue culture plates (Corning Costar TC‐Treated 24‐Well Plate, Sigma Aldrich), left overnight at 4 °C and washed twice with PBS. A qualitative assessment of dispersions was performed using Fiji (ImageJ: http://imagej.nih.gov/ij, v1.53t).

### Human Neural Stem Cell Culture

hNSCs (Merck Millipore, SCC008) were maintained as monolayer cultures seeded at an initial density of 10 × 10^4^ cells per well onto laminin (10 µg mL^−1^ in PBS, pH 7.4; Sigma Aldrich) coated six‐well plates (Corning) containing NeuroCult NS‐A Proliferation Kit (Human: consisting of NeuroCult NS‐A Basal Medium and NeuroCult NS‐A Proliferation Supplement, STEMCELL Technologies), and further supplemented with heparin (2 µg mL^−1^, Sigma Aldrich), epidermal growth factor (EGF, 20 ng mL^−1^; Peprotech, Lonza) and basic fibroblast growth factor (bFGF, 20 ng mL^−1^; Peprotech, Lonza). Cell cultures were maintained in a 5% CO_2_ humidified incubator at 37 °C, and the culture medium was refreshed every 2–3 days with half‐volume medium changes.

hNSCs were passaged for subculture every 5–7 days by digesting in TrypLE Select (Gibco, ThermoFisher Scientific) for 3 min in a 5% CO_2_ humidified incubator at 37 °C. Digested cultures were triturated to single cells following the addition of prewarmed culture medium, centrifuged at 190 × g for 3 min, resuspended in a fresh prewarmed culture medium, and re‐plated as above. Experimentation involving this cell line was approved for use by the University of Wollongong's Human Research Ethics Committee (HE14/049).

### Optimisation of Barium Titanate Nanoparticle‐Laden Spheroid Formation

hNSCs were seeded at 20 000 cells well^−1^ onto either BTNP‐PDL (1000, 500, and 250 µg mL^−1^), nondispersed BTNPs (1000, 500, and 250 µg mL^−1^) or PDL (500 µg mL^−1^) in PBS within a 24‐well tissue culture plate (Corning Costar TC‐Treated 24‐Well plate, Sigma Aldrich). Cells were then cultured at 37 °C in a humidified 5% CO_2_ incubator for 7 days in proliferation medium (NeuroCult NS‐A Proliferation Medium Kit), heparin, EGF, and bFGF. The proliferation medium was changed every 3 days. Differentiation of spheroids was performed by culturing for a further 5 days in neural differentiation media comprising Neurobasal Media (50% final volume; Gibco, ThermoFisher Scientific) and DMEM/F12 media (50% final volume; Gibco, ThermoFisher Scientific), and supplemented with 1% (v/v) B‐27 supplement (Gibco, ThermoFisher Scientific, 17504044), 0.5% (v/v) N2 supplement‐A (STEMCELL Technologies, 07152), L‐Glutamine at 200 mm final concentration (Gibco, ThermoFisher Scientific, 25030081) and brain‐derived neurotrophic factor (BDNF, 50 ng mL^−1^, Peprotech, 450‐02). The differentiation medium was changed every 3 days.

### Stimulation of Barium Titanate Nanoparticle‐Laden Spheroids

For stimulation experiments, neural spheroids were transferred to laminin‐coated 4‐well chamber slides (Nunc Lab‐Tek II Chamber Slide system, ThermoFisher Scientific; area per well: 1.7 cm^2^) and allowed to adhere for 24 h before the media was changed to neural differentiation media (as described above). Following the media change, ultrasound was applied to neural spheroids with and without BTNPs using an underwater sonicator device (LABEC; Ultrasonics Australia, FXP4, 1.1 L; transducer power: 50 W, operating frequency 40 kHz) filled with sterile water treated with Penicillin/Streptomycin antibiotic (1:1000). The neural spheroids were stimulated for 180 s followed by 1 h of recovery, repeated five times per day, for 5 days in total, whilst maintained in a 5% CO_2_ humidified incubator at 37 °C.

### Live‐Cell Confocal Microscopy of Barium Titanate Nanoparticle Loading of Spheroids

BTNPs were visualized with PLL‐FITC (Sigma Aldrich, P3543), which is the naturally occurring enantiomer of PDL. Whilst not the preferred choice for coating BTNPs for USPZ stimulation of 3D neural tissue due to being less resistant to enzymatic degradation, PLL‐FITC is commercially available and suitably durable for shorter‐term assessment of BTNP loading of spheroids. BTNPs were coated with PLL‐FITC by sonicating with PLL‐FITC at 1 µg mL^−1^ for 6 h. The labeled BTNPs were then diluted 1:2 and coated onto the surface of a 24‐well tissue culture plate (Corning Costar TC‐Treated 24‐Well plate, Sigma Aldrich), left overnight, and then washed twice with PBS. Vybrant CM‐Dil Cell Labelling solution (V22888, Invitrogen, ThermoFisher Scientific, Ex: 553 nm, Em: 560 nm, 1:200 in proliferation medium) was used to counter stain plasma membranes, intracellular membranes, liposomes, and lipoproteins of live hNSCs. Cells were incubated overnight at 37 °C followed by imaging with a Leica TCS SP5 II confocal microscope (Leica Microsystems). Image analysis was conducted using Fiji (ImageJ).

### Scanning Electron Microscopy with Energy Dispersive X‐Ray Spectroscopy

A FEI Helios Nanolab 650 dual‐FIB‐SEM were used to acquire photomicrographs of laminin‐coated glass slide immobilized neural spheroids, with or without incorporated BTNPs. Briefly, samples for FIB‐SEM imaging were prepared by immersion in liquid nitrogen, followed by dehydration and bulk platinum coating. The imaging site was then prepared with an additional injection of platinum deposition to provide strength to the area to be milled. A cross‐sectional area of immobilized neural spheroids was exposed by focused ion beam trenching, and the milling process was continuously monitored by SEM imaging to reveal an area of interest for subsequent high‐resolution SEM imaging and energy dispersive spectroscopic (EDS) analysis.

### Cell Viability Assay

Tissue constructs were incubated with Calcein AM (5 µg mL^−1^, ThermoFisher Scientific) at 37 °C for 45 min followed by incubation in propidium iodide (PI, 5 µg mL^−1^, ThermoFisher Scientific) for 10 min. Tissues were then washed and imaged in DMEM/F12 (Gibco, ThermoFisher Scientific). Images were acquired using a Leica TCS SP5 II confocal microscope (Leica Microsystems). Quantification of live and dead cells was performed using Fiji (ImageJ) and applying a threshold to remove noise.

### Cell Proliferation Assay

PrestoBlue reagent (Molecular Probes, Invitrogen, ThermoFisher Scientific, A13261) was added directly to cells in culture medium at 1:10 dilution for 2 h at 37 °C in a humidified 5% CO_2_ incubator. To correct for background florescence, control wells containing only cell media were incubated with PrestoBlue reagent under the same conditions. Following incubation, 100 µL of supernatant for each sample was loaded onto a 96‐well flat‐bottom PS microplate (Greiner Bio‐One, 655101). Fluorescence intensity was measured by a spectrophotometer (Ex: 535 nm, Em: 615 nm, POLARstar Omega, BMG Labtech). Following assessment, cultures were rinsed in DMEM/F12 and returned to the incubator with fresh neural differentiation media for ongoing culture. Background fluorescence was subtracted, and fluorescence was expressed at the percentage increase from day 1 to day 5 of ultrasound stimulation.

### Immunocytochemistry

Spheroids and derivative tissues were fixed with 3.7% paraformaldehyde for 30 min at room temperature, followed by concurrent block and permeabilization with 5% (v/v) goat serum (Sigma Aldrich) and 0.3% (v/v) Triton X‐100 (Sigma Aldrich) overnight at 4 °C. Samples were then incubated with primary antibodies in 5% (v/v) goat serum (Sigma Aldrich) at the following dilutions: anti‐NES (1:200, Merck, MAB5326), anti‐SOX2 (SOX2, 1:200, Merck A5603), anti‐VIM (1:200, Merck, MAB3400), anti‐KI67 (MKI67, 1:200, Abcam, ab15580), anti‐TUBB3 (clone TUJ1, 1:1000, Covance, MMS‐435P), anti‐GFAP (1:1000, Merck, AB5840), anti‐MAP2 (1:1000, Merck, MAB3418), anti‐SYP (1:250, Abcam, ab32127). Samples were washed three times in 0.1% (v/v) Triton X‐100 in PBS for 10 min at room temperature prior to incubation with species‐specific Alexa Fluor conjugated secondary antibodies (Alexa Flour 488, A28175; Alexa Flour 546, A‐11010) in 5% goat serum in PBS for 2 h at room temperature. Samples were then rinsed in 0.1% Triton X‐100 in PBS (v/v) prior to nuclear staining with 4′,6‐diamidino‐2‐phenylindole (DAPI, 1:1000, Invitrogen, ThermoFisher Scientific) for 10 min at room temperature. Secondary antibody‐only controls were applied to confirm no nonspecific secondary antibody labeling. A Leica TSC SP5 II confocal microscope (Leica Microsystems) was used for image acquisition together with Leica Application Suite AF (LAS AF) software. Sequential imaging was applied to avoid spectral bleed‐through.

### F‐actin Labeling

Tissues were fixed with 3.7% PFA for 30 min at room temperature, followed by permeabilization for 15 min with 0.1% Triton X‐100 in PBS at 4 °C. They were then incubated with Alexa Fluor 488‐conjugated Phalloidin (1:40, Invitrogen, ThermoFisher Scientific, A12357) with 1% BSA in PBS for 90 min at room temperature, washed twice with PBS, and nuclei were counterstained with DAPI (1:1000) in PBS for 10 min at room temperature. A Leica TSC SP5 II confocal microscope (Leica Microsystems) was used for image acquisition together with Leica Application Suite AF (LAS AF) software.

### Live Cell Calcium Imaging

Tissues were incubated in Fluo‐4 AM in Dimethyl Sulfoxide (1 µm, ThermoFisher Scientific) in fresh neural differentiation culture medium for 45 min at 37 °C in a humidified 5% CO_2_ incubator. They were then washed in 4‐(2‐hydroxyethyl)1‐piperazineethanesulfonic acid (HEPES)‐buffered Hank's balanced salt solution (137 mm NaCl, 5.6 mm D‐Glucose, 10 mm HEPES, 1.5 mm CaCl_2_, 5.4 mm KCl_2_, 0.4 mm KH_2_PO_4_, 0.5 mm MgCl_2_, 0.4 mm MgSO_4_, 0.3 mm Na_2_HPO_4_, and 4 mm NaHCO_3_, pH 7.4). After 10 min, time‐lapse imaging of spontaneous intracellular calcium flux was performed using a Leica TSC SP5 II confocal microscope (Leica Microsystems) with LAS AF software. To augment intracellular calcium flux, GABA_A_ antagonist bicuculline was added, and the cultures were imaged for a further 5 min with images taken at 2 s intervals over a period of 150–300 s. Images were assessed using FluroSNNAP software.^[^ ^16^ ^]^ For each sample, 80 cell bodies were manually defined as regions of interest (ROIs). The amplitude (ΔF/F_0_) per ROI over the time series was calculated and normalized by subtracting from each value the mean of the lower 50% of the previous 10 s values and dividing by the mean of the lower 50% of the previous 10 s values. The mean oscillation period was automatically calculated and averaged over all ROIs in a sample to give mean frequency (Hz). The inferred functional connectivity (expressed as mean global connectivity in arbitrary units (a.u.) was calculated to provide information regarding the temporal interaction of neurons. Mean functional connectivity was calculated using a partial correlation model and accepted as significant at *p* < 0.05. Network ensembles were defined as coactivation of a group of neurons in a high‐activity frame working in concert, rather than as single neurons. Ensembles were determined through binarized activity trace (threshold spike probability) at three standard deviations and reshuffling active neurons 1000 times by randomly transposing intervals of activity within each cell to calculate groups of statistically significant co‐active neurons (*p* < 0.05) (Miller et al., 2014). The firing rate of ensembles (s) was plotted and analyzed.

### Statistical Analyses

Data are represented as mean ± standard deviation (SD) of three independent experiments unless otherwise indicated. Evaluation of differences in means was determined by an independent‐samples t‐test or a two‐way ANOVA with Bonferroni post hoc test for multiple comparisons. Statistical assumptions were tested by normality of data distribution (Kolmogorov–Smirnov test) and homogeneity of variance (Levene's test). If assumptions were violated, a Mann–Whitney test was performed as a nonparametric equivalent of the independent samples *t*‐test. If homogeneity of variance was satisfied (*p* > 0.05), the statistical significance of two‐way ANOVA was set at *p* < 0.05. If homogeneity of variance was not satisfied (*p* < 0.05), the statistical significance of two‐way ANOVA was set at *p* < 0.01. GraphPad Prism 5 (GraphPad Software, Inc., La Jolla, CA, USA) and SPSS (IMB version 21.0 Inc., NY, USA) software were used for graphing and statistical analyses.

## Conflict of Interest

The authors declare no conflict of interest.

## Supporting information

Supporting Information

Supplemental Video 1

Supplemental Video 2

Supplemental Video 3

## Data Availability

The data that support the findings of this study are available from the corresponding author upon reasonable request.
